# Factors associated with drug prescribing practices in long-term care patients with cognitive impairment

**DOI:** 10.1007/s41999-020-00331-0

**Published:** 2020-05-25

**Authors:** Violetta Kijowska, Ilona Barańska, Katarzyna Szczerbińska

**Affiliations:** Laboratory for Research on Aging Society, Department of Sociology of Medicine, Chair of Epidemiology and Preventive Medicine, Jagiellonian University Medical College, Kopernika 7a Street, 31-034 Kraków, Poland

**Keywords:** Cognitive impairment, Antipsychotics, Antianxiety agents, Nursing homes, Residential facilities, InterRAI-LTCF tool

## Abstract

**Purpose:**

To examine factors associated with prescribing anti-dementia medicines (ADM), atypical antipsychotics (A-APM), typical antipsychotics (T-APM), anxiolytics and other psychostimulants (OP) in the residents of long-term care institutions (LTCIs).

**Methods:**

A cross-sectional survey of a country-representative sample of randomly selected LTCIs in Poland, conducted in 2015–2016. First, we identified 1035 residents with cognitive impairment (CI) among all 1587 residents. Next, we randomly selected 20 residents from each institution. Study sample consists of 455 residents with CI: 214 recruited from 11 nursing homes and 241 from 12 residential homes. We used InterRAI-LTCF questionnaire and drug dispensary cards administered on the day of data collection to assess use of drugs. Multiple correspondence analysis (MCA), descriptive and logistic regression analyses were performed.

**Results:**

The residents were treated with ADM (13.4%), OP (14.3%), antipsychotics (46.4%) including A-APM (24.2%) and T-APM (27.9%), and anxiolytics (28.4%). Hydroxyzine was used most often among anxiolytics (71.3%). Prescribing of ADM was more likely in Alzheimer’s disease (OR = 4.378; 95%CI 2.173–8.823), while OP in other dementia (OR = 1.873; 95%CI 1.007–3.485). Administration of A-APM was more likely in older residents (OR = 1.032, 95%CI 1.009–1.055), and when delusions appeared (OR = 2.082; 95%CI 1.199–3.613), while there were no neuropsychiatric factors increasing the odds of T-APM use. Prescribing of anxiolytics was less likely in moderate CI (by 47.2%) than in residents with mild CI.

**Conclusion:**

Current practices of prescribing psychotropics are inadequate in Polish LTCIs, especially in terms of use of T-APM and hydroxyzine. More attention should be given to motivate physicians to change their prescribing practices.

**Electronic supplementary material:**

The online version of this article (10.1007/s41999-020-00331-0) contains supplementary material, which is available to authorized users.

## Key summary points

**Aim:**

To examine factors related to resident's characteristics which are associated with prescribing anti-dementia medicines, atypical antipsychotics, typical antipsychotics, anxiolytics and other psychostimulants in the individuals with cognitive impairment residing in long-term care institutions.

**Findings:**

There are still many long-term care (LTC) residents who receive medications that are not recommended or even contraindicated in dementia. Despite existing clinical recommendations for treatment of cognitive impairment and neuropsychiatric symptoms, the physicians taking care for LTC residents do not follow them properly.

**Message:**

Since ca. 70% of LTC residents have cognitive impairment, all physicians taking care of these patients should be trained in clinical guidelines of dementia treatment.

**Electronic supplementary material:**

The online version of this article (10.1007/s41999-020-00331-0) contains supplementary material, which is available to authorized users.

## Introduction

Dementia is a major neurocognitive disorder predominated by symptoms of cognitive function impairment, and often accompanied with neuropsychiatric symptoms (NPSs) [1, 2]. Pharmacological treatment of dementia essentially focuses on the treatment of these two groups of symptoms through the use of anti-dementia (ADM) and antipsychotic medicines (APM). During the last 2 decades, treatment of this disorder evolved towards the use of ADM (e.g. donepezil, rivastigmine, memantine) and atypical antipsychotic drugs (A-APM) (e.g. quetiapine, risperidone, olanzapine), which gradually replace nootropic drugs (e.g. piracetam, vinpocetine), typical antipsychotics (T-APM) (e.g. haloperidol, promazine) and benzodiazepines. The latter, due to side effects, are currently not recommended for older people [3]. In light of ongoing discussions on the excessive use of medicines and the accumulation of adverse effects in older adults, a rational approach to treating these disorders in long-term care (LTC) residents is particularly relevant. It is also important, because the prevalence of cognitive impairment (CI) in these settings is very high, reaching about 60–70% in most European [4] and Northern American countries [5], as well as in Poland [6]. Patients with CI are the main group of residents taking psychotropic drugs in nonpsychiatric institutions. The way they are treated affects not only their quality of life and health status [7–9], but also the economic results of the institutions they are residing in [10, 11].

In Poland, there are two types of LTC institutions (LTCIs): residential homes (RH) served by off-site general practitioners (GP) and nursing homes (NH) with physicians (with various specializations) employed for 24 h per 7 days a week. These facilities are organized and funded on a different basis (described elsewhere) [12], where prescribing and administering of medicines are organized differently (supplemental Table 1). Patients with CI may be equally referred to RHs and NHs, however, due to the diverse range of services provided in these facilities, treatment of CI may differ between them.

The main aim of this study was to describe the current practices of pharmacological treatment of residents with CI in LTCIs, with a focus on:

- identifying patterns of inappropriate prescribing in reference to the level of CI and the type of setting (NH and RH), and
- identifying predictors of prescribing specific drugs classes.
  To achieve these goals, we have conducted:
- a comparison of use of certain drug classes in light of data from international studies;
- a comparison of prescription of anti-dementia medications (ADM) and treatment with other psychostimulants (OP);
- a comparison of prescription of typical (T-APM) and atypical antipsychotic medicines (A-APM);
- a logistic regression analysis to find factors associated with administering ADM, T-APM, A-APM, anxiolytics and OP;
- a multiple correspondence analysis (MCA) to check which classes of psychotropic drugs are jointly prescribed in LTC residents depending on the resident’s age, the level of CI, and the type of facility, they reside.

## Methods

### Settings and participants

The study was performed in 2015–2016 within 23 LTC institutions in Poland, randomly selected in all 6 regions in terms of size, status, geographical region, number of beds and facility type: NH, which is similar to a skilled nursing facility, and RH—a residential facility referring to the classification proposed by experts’ panel in JAMDA in 2015 [13]. A detailed description of inclusion criteria to the study [6] and a comparison of both types of the facilities have been published elsewhere [12]. The study received approval from the relevant University Ethics Committee (decision No. 122.6120.31.2015).

### Study design

From a total of 1587 residents admitted to 23 facilities, we excluded 93 individuals who were unable to express any cognitive or neuropsychiatric symptoms (NPSs) due to indiscernible consciousness or coma. In the first stage of the study, we identified 1035 residents with CI based on Cognitive Performance Scale (CPS) [14] score with a cutoff of 2 points. Next, 20 residents with CI were randomly selected from each facility and included in a study group of 455 residents who were then studied with the interRAI-LTCF questionnaire (interRAI Long-Term Care Facilities Assessment System questionnaire)—a validated and widely used tool enabling comprehensive geriatric assessment of people receiving LTC services [15]. LTC residents were assessed by regular staff at each institution—a nurse or a psychologist—who passed standardized training performed by one researcher as specified in the user’s manual of the interRAI-LTCF tool [16]. The assessments were done mainly on the basis of a 3-day observation of the residents, and supported (if necessary) by data obtained from medical files, family members or other staff. The study protocol and comprehensive characteristics of the study group has been described in detail elsewhere [6, 12].

### Measurements

A seven-point CPS scale, embedded in interRAI-LTCF instrument, was used to assess CI as mild (CPS = 2), moderate (CPS = 3–4) or severe (CPS = 5–6). This is a widely used scale [17, 18], which demonstrated a high correlation with the Mini-Mental State Examination (MMSE) [14, 19], the Montreal Cognitive Assessment (MoCA) [17], and the Test for Severe Impairment, nursing judgments of disorientation, and neurological diagnoses of Alzheimer's disease (AD) and other dementias [14] in LTC residents. A seven-point Activities of Daily Living Hierarchy scale (ADLh), measuring functional performance based on four items: personal hygiene, locomotion, toilet use and eating, categorized the LTC residents as independent (ADLh = 0–1); moderately dependent (ADLh = 2–3), and severely dependent (ADLh = 4–6) [20]. We also applied the four-item Aggressive Behavior Scale (ABS) to measure verbal and physical abuse, socially inappropriate behavior, and resistance to care, as well as we considered each of these variables separately. The ABS ranges from 0 to 12, where a higher score indicates a greater frequency of aggressive behavior [21]. Depression symptoms were evaluated using the seven-item Depression Rating Scale (DRS), which indicates probable depression, when its score is 3 and higher [22].

The analyses in this paper were conducted with focus on comparing drug use by residents in NHs and RHs. Data were collected from drug dispensary cards on the day of data collection. We used the Anatomical Therapeutic Chemical (ATC) classification [23] to group medicines as following: anti-dementia medicines (ADM) (N06DA, N06DX), other psychostimulants (OP) (N06BX), atypical antipsychotics (A-APM) (N05AH, N05AX), typical antipsychotics (T-APM) (N05AA, N05AB, N05AD, N05AF, N05AL), and anxiolytics (N05BA, N05BB, N05CD, N05CF).

### Statistical analysis

A detailed list of medications with their use by residents depending on the type of institution (RH or NH) is shown in Table 2. In the Table 1, we presented relationships between the use of selected ATC groups of drugs and the resident characteristics (differences were assessed using the Chi^2^ test). In our analyses, we carefully selected the variables including: demographic factors (age, gender); type of setting (due to differences in care organization and access to physician); severity of CI and level of ADL; factors which may trigger use of psychotropic medicines such as: psychotic symptoms (hallucinations, delusions), behavior problems (agitation, wandering, verbal and physical abuse, resistance to care); depression, psychiatric illness, Alzheimer’s disease and other dementia, which may cause indications for use of these drugs; and use of restrictive devices as they might be associated with administering pharmacologic restraints. All these variables were checked consecutively in the univariate analysis and logistic regression analysis for their associations with the use of drugs from particular ATC classes to determine residents’ characteristics associated with their prescribing (Table 3). The variable of ADL level was excluded due to collinearity with the CPS level. Some associations occurred to be not straightforward, but more complex, and are presented as interactions between variables. They are result of careful following the principles of performing logistic regression analysis. The way how they were calculated had been shown in Table 3, and described how they should be interpreted in the results section.

**Table 1:** The use of medications in LTC residents with cognitive impairment in relation to their characteristics, health issues, behavioral symptoms and severity of cognitive impairment

Characteristics of LTC residents	Total sample *n* = 455 %(*n*)	Number of residents treated with drugs from certain ATC classes									
		Anti-dementia medicines		Other psychostimulants		Typical antipsychotics		Atypical antipsychotics		Anxiolytics	
		13.4% (61)		14.3% (65)		27.9% (127)		24.2% (110)		28.4% (129)	
		% (*n*)	*p* value	% (*n*)	*p* value	% (*n*)	*p* value	% (*n*)	*p* value	% (*n*)	*p* value
Facility type (*n* = 455)											
Nursing homes (NHs)	**47.0 (214)**	8.4 (18)	**0.003**	16.8 (36)	0.145	31.8 (68)	0.083	21.0 (45)	0.139	27.1 (58)	0.578
Residential homes (RHs)	**53.0 (241)**	17.8 (43)		12.0 (29)		24.5 (59)		27.0 (65)		29.5 (71)	
*Resident characteristics*											
Gender (*n* = 455)											
Female	**70.1 (319)**	15.4 (49)	0.061	15.7 (50)	0.195	28.2 (90)	0.826	24.5 (78)	0.833	28.2 (90)	0.920
Vs male	**29.1 (136)**	8.8 (12)		11.0 (15)		27.2 (37)		23.5 (32)		28.7 (39)	
Age of the resident (*n* = 455)											
< 65 years	**15.8 (72)**	8.3 (6)		9.7 (7)		31.9 (23)		16.7 (12)		25.0 (18)	
65–74 years	**17.1 (78)**	9.0 (7)	0.117	10.3 (8)	0.212	34.6 (27)	0.278	14.1 (11)	**0.021**	30.8 (24)	0.770
75–84 years	**32.6 (148)**	18.2 (27)		14.2 (21)		27.0 (40)		28.4 (42)		30.4 (45)	
≥ 85 years	**34.5 (157)**	13.4 (21)		18.5 (29)		23.6 (37)		28.7 (45)		26.8 (42)	
ADL dependency^a^ (*n* = 454)											
No	**11.5 (52)**	11.5 (6)		11.5 (6)		28.8 (15)		25.0 (13)		34.6 (18)	
Moderate	**27.5 (125)**	15.2 (19)	0.763	12.8 (16)	0.641	24.8 (31)	0.649	20.8 (26)	0.614	26.4 (33)	0.527
Severe	**61.0 (277)**	13.0 (36)		15.5 (43)		29.2 (81)		25.3 (70)		27.8 (77)	
Cognitive impairment (CPS)^b^ (n = 455)											
Mild	**36.0 (164)**	10.4 (17)		12.2 (20)		25.0 (41)		24.4 (40)		31.7 (52)	
Moderate	**22.0 (100)**	16.0 (16)	0.342	19.0 (19)	0.291	26.0 (26)	0.361	22.0 (22)	0.836	21.0 (21)	0.161
Severe	**42.0 (191)**	14.7 (28)		13.6 (26)		31.4 (60)		25.1 (48)		29.3 (56)	
*Chronic diseases and symptoms of diseases*											
Alzheimer's disease (*n* = 450)											
Yes	**12.2 (55)**	40.0 (22)	** < 0.001**	14.5 (8)	0.982	25.5 (14)	0.654	29.1 (16)	0.392	36.4 (20)	0.178
vs No	**87.8 (395)**	9.9 (39)		14.4 (57)		28.4 (112)		23.8 (94)		27.6 (109)	
Other dementia (*n* = 450)											
Yes	**60.2 (271)**	11.1 (30)	0.114	17.0 (46)	**0.040**	29.5 (80)	0.452	26.6 (72)	0.087	27.7 (75)	0.656
vs No	**39.8 (179)**	16.2 (29)		10.1 (18)		26.3 (47)		19.6 (35)		29.6 (53)	
Symptoms of depression (DRSs)^c^ (n=443)											
Yes	**38.6 (171)**	12.9 (22)	0.741	15.8 (27)	0.454	29.8 (51)	0.551	25.7 (44)	0.599	30.4 (52)	0.521
vs No	**61.4 (272)**	14.0 (38)		13.2 (36)		27.2 (74)		23.5 (64)		27.6 (75)	
Psychiatric diseases other than depression and dementia (*n* = 452)											
Yes	**5.3 (24)**	16.7 (4)	0.549	12.5 (3)	0.787	37.5 (9)	0.292	29.2 (7)	0.571	29.2 (7)	0.925
vs No	**94.7 (428)**	13.3 (57)		14.5 (62)		27.6 (118)		24.1 (103)		28.3 (121)	
Hallucinations (*n* = 452)											
Yes	**16.2 (73)**	17.8 (13)	0.212	12.3 (9)	0.585	31.5 (23)	0.479	27.4 (20)	0.506	37.0 (27)	0.073
vs No	**83.8 (379)**	12.4 (47)		14.8 (56)		27.4 (104)		23.7 (90)		26.6 (101)	
Delusions (*n* = 451)											
Yes	**24.4 (110)**	23.6 (26)	** < 0.001**	11.8 (13)	0.412	29.1 (32)	0.757	32.7 (36)	**0.019**	29.1 (32)	0.896
vs No	**75.6 (341)**	10.3 (35)		15.0 (51)		27.6 (94)		21.7 (74)		28.4 (97)	
Agitation (*n* = 452)											
Yes	**30.3 (137)**	17.5 (24)	0.099	10.9 (15)	0.170	34.3 (47)	**0.037**	23.4 (32)	0.749	35.0 (48)	**0.044**
vs No	**69.7 (315)**	11.7 (37)		15.9 (50)		24.8 (78)		24.8 (78)		25.7 (81)	
*Social functioning*											
Regular contact with family members or relatives (*n* = 452)											
Yes	**58.4 (264)**	14.0 (37)	0.702	14.4 (38)	0.992	29.5 (78)	0.348	26.1 (69)	0.291	26.1 (69)	0.222
Vs No	**41.6 (188)**	12.8(24)		14.4 (27)		25.5 (48)		21.8 (41)		31.4 (59)	
Behavior problems (in the last 3 days):											
Wandering (*n* = 450)											
Yes	**22.7 (102)**	21.6 (22)	**0.005**	14.7 (15)	0.874	33.3 (34)	0.173	31.4 (32)	0.055	30.4 (31)	0.661
Vs No	**77.3 (348)**	10.9 (38)		14.1 (49)		26.4 (92)		22.1 (77)		28.2 (98)	
Verbal abuse (*n* = 451)											
Yes	**26.8 (121)**	11.6 (14)	0.512	15.7 (19)	0.577	28.9 (35)	0.777	22.3 (27)	0.577	36.4 (44)	**0.027**
Vs No	**73.2 (330)**	13.9 (46)		13.6 (45)		27.6 (91)		24.8 (82)		25.8 (85)	
Physical abuse (*n* = 448)											
Yes	**16.3 (73)**	19.2 (14)	0.113	15.1 (11)	0.835	32.9 (24)	0.278	23.3 (17)	0.820	41.1 (30)	**0.010**
Vs No	**83.7 (375)**	12.3 (46)		14.1 (53)		26.7 (100)		24.5 (92)		26.1 (98)	
Socially inappropriate behavior (*n* = 451)											
Yes	**32.8 (148)**	14.2 (21)	0.699	16.9 (25)	0.251	32.4 (48)	0.137	23.6 (35)	0.857	32.4 (48)	0.209
Vs No	**67.2 (303)**	12.9 (39)		12.9 (39)		25.7 (78)		24.4 (74)		26.7 (81)	
Resistance to care (*n* = 453)											
Yes	**32.7 (148)**	13.5 (20)	0.983	14.2 (21)	0.946	34.5 (51)	**0.028**	19.6 (29)	0.105	30.4 (45)	0.526
Vs No	**67.3 (305)**	13.4 (41)		14.4 (44)		24.6 (75)		26.6 (81)		27.5 (84)	
Aggressive behavior (ABS)^d^ (*n* = 448)											
Yes, any sign (≥ 1)	**49.6 (222)**	13.1 (29)	0.839	14.4 (32)	0.939	31.5 (70)	0.071	23.4 (52)	0.657	30.2 (67)	0.455
Vs No signs (0)	**50.4 (226)**	13.7 (31)		14.2 (32)		23.9 (54)		25.2 (57)		27.0 (61)	
*Restrictive devices*											
Full bed rails (*n* = 452)											
Yes	**57.1 (258)**	13.6 (35)	0.833	14.7 (38)	0.808	30.6 (79)	0.134	24.8 (64)	0.692	29.8 (77)	0.406
Vs No	**42.9 (194)**	12.9 (25)		13.9 (27)		24.2 (47)		23.2 (45)		26.3 (51)	
Trunk restraints (*n* = 446)											
Yes	**5.4 (24)**	20.8 (5)	0.229	12.5 (3)	0.767	37.5 (9)	0.301	16.7 (4)	0.388	29.2 (7)	0.898
Vs No	**94.6 (422)**	12.8 (54)		14.7 (62)		27.7 (117)		24.4 (103)		28.0 (118)	
Chair preventing rising^e^ (*n* = 447)											
Yes	**12.3 (55)**	12.7 (7)	0.912	18.2 (10)	0.413	38.2 (21)	0.071	40.0 (22)	**0.004**	23.6 (13)	0.423
Vs No	**87.7 (392)**	13.3 (52)		14.0 (55)		26.5 (104)		22.2 (87)		28.8 (113)	

Chi2 test to show relationships between the use of selected groups of drugs and the resident and facility characteristics. Values for which *p* < 0.05 are highlighted in bold

Missing values: ADL dependency—1; Alzheimer’s disease—5; dementia—5; symptoms of depression (DRS)—12; psychiatric diseases other than depression and dementia—3, hallucinations—3, delusions—4; agitation—3; regular contact with family members or relatives—3; behavior problems: wandering—5; verbal abuse—4; physical abuse—7; inappropriate behavior—4, resistance to care—2; Aggressive behavior (ABS)—7; restrictive devices: full bed rails on all open sides of bed—3; trunk restrains—9; chair prevent rising—8

^a^Activities of daily living (ADL) self-performance hierarchy scale: minimal (0–1), moderate (2–3), and severe ADL limitations (4–6)

^b^Cognitive performance scale (CPS): mild (2), moderate (3–4), and severe impairment (5–6)

^c^Symptoms of depression- depression rating scale (DRS): no depressive disorders (0–2), minor-to-major depressive disorders (3–14)

^d^Aggressive Behavior Scale (ABS): no signs of aggressive behavior (0), Yes, any sign of aggressive behaviors (1–12)

^e^Chair preventing rising: armchair with strips to prevent uncontrolled rising or falling

**Fig. 1:**
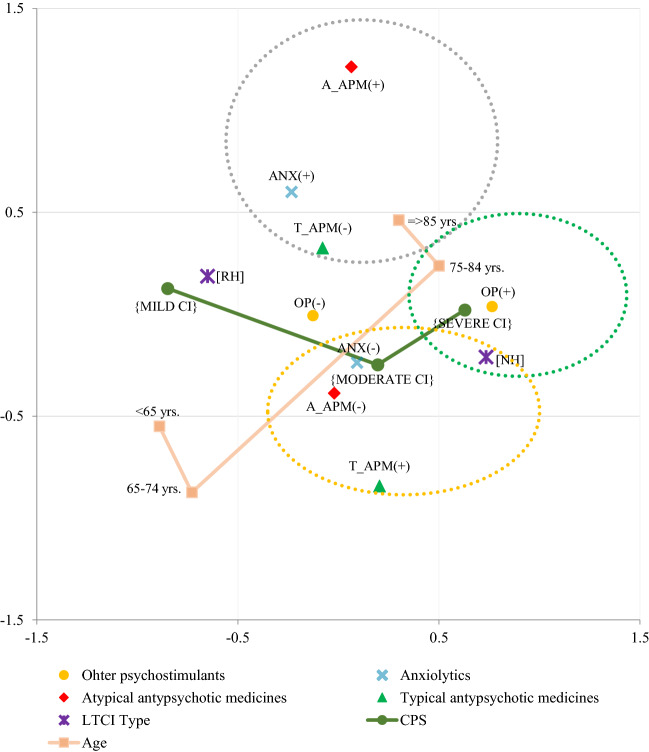
Multiple correspondence analysis plot for two dimensions of the use of different medications in LTC residents with cognitive impairment (CI) in relation to the facility type, resident’s age and the level of CI

The logistic regression analyses provided information on the predictors of prescribing a single drug classes. However, in the reality, these drugs are often used together. Therefore, we have also carried out a Multiple Correspondence Analysis (MCA) which belongs to a family of descriptive methods, that allows to investigate the correlation between several categorical interdependent variables, measuring the level of inertia among them. It provides a general view of relationships between variables when simple cross-tabulations of numerous variables become complex and difficult to interpret. We used this analysis to explore the relationships between the use of drugs and the variables characterizing the residents (Fig. 1). First, we considered information about use of psychotropics from particular ATC classes as well as basic characteristics of the resident presented in Table 1 (age, gender, type of facility, ADL, CPS). Gender and ADL variables had very low values of discriminatory measures on both dimensions, so they were removed from the analysis. We also excluded ADM from the MCA, because this variable was strongly correlated with presence of Alzheimer’s disease. Finally, we identified two dimensions (axes), and seven variables (A-APM, T-APM, anxiolytics, OP, LTCI type, level of CI and resident’s age), the most correlated with each dimension. In MCA, the squared correlations between variables and the dimensions are used as coordinates. The distance between any row points or column points gives a measure of their similarity (or dissimilarity). The variables the most related to each other are focused in a group together, whilst dissimilarity, on the other hand, results in a distance. Negatively correlated variables are positioned on opposite sides of the plot origin (opposed quadrants).

By applying MCA, we explored the correlation between use the certain psychotropic drug classes (A-APM, T-APM, OP and anxiolytics) in controlling certain resident’s characteristics such as: age, CPS level and facility type. The figure above helps to identify seven variables (CI level, resident’s age, type of facility, use of drugs from ATC classes: A-APM, T-APM, OP and anxiolytics) that are the most correlated with each dimension. The squared correlations between variables and the dimensions are used as coordinates. The distance between any row points or column points gives a measure of their similarity (or dissimilarity). Variables that make up the group are focused in a group together, whilst dissimilarity, on the other hand, results in a distance. Negatively correlated variable categories are positioned on opposite sides of the plot origin (opposed quadrants). Legend: *NH *nursing home, *RH* residential home, *mild CI *mild cognitive impairment, *moderate CI *moderate cognitive impairment, *severe CI *severe cognitive impairment, *T_APM *typical antipsychotics, *A_APM *atypical antipsychotics, *OP* other psychostimulants, *ANX* anxiolytics. Plus (+) means that the patient received a particular drug, whereas minus (−) means no usage of particular drug.

Analyses were performed with SPSS 25 for Windows. Differences were considered statistically significant if *p* value was less than 0.05.

## Results

### Resident characteristics and use of drugs

The residents with CI in our study were diagnosed for AD (ICD-coded with G.30) (12.2%) or other dementia (60.2%) (ICD-coded with F01, F02 or F03), had symptoms of depression (38.6%) or psychiatric diseases other than depression and dementia (5.3%). More than 70% residents were female, and 42.0% had severe CI, while 88.5% were moderate-to-severely dependent in ADL (Table 1). They were treated with ADM (13.4%), OP (14.3%), any antipsychotics (46.4%) and anxiolytics (28.4%). We found that ADM were significantly more often administered to RH residents (17.8%) than to NH patients (8.4%), while prescribing of A-APM was more frequent in older patients at the age 75 years and older (approx. 30%). There was no impact of gender, neither ADL dependency, nor level of CI on frequency of medication use. However, some health conditions (AD, other dementia), symptoms (delusions, agitation), and behavior problems (wandering, verbal and physical abuse, resistance to care) showed association with significantly higher use of the aforementioned medicines. The use of chair preventing rising was associated with almost twice the higher use of A-APM.

The comparison between types of facilities did not show significant differences in use of certain drugs classes or substances with the exception of donepezil, rivastigmine, and olanzapine, which were more frequent in RH residents with CI (as shown in Table 2). It is worth noting that some of the drugs were prescribed more often than the others, e.g. donepezil in 7.7% of residents (among ADM), quetiapine in 15.4% individuals (among A-APM), perazine (10.1%), promazine (7.9%) and haloperidol (7.5%) (among T-APM) and hydroxyzine (in 20.2% residents) (among anxiolytics).

**Table 2:** The use of certain medicine substances and drug classes in residents with cognitive impairment in relation to the type of LTC institution

Group	Sub-group	ATC code^a^	List of drugs				
			Name	Total sample % (*n*)	Nursing home (NH) % (*n*)	Residential home (RH) % (*n*)	*p* value
Anti-dementia medicines 13.4% (61)	Anti-dementia drugs (N06D)	N06DA02	*Donepezil*	7.7 (35)	4.7 (10)	10.4 (25)	**0.023**
		N06DA03	*Rivastigmine*	2.6 (12)	0.5 (1)	4.6 (11)	**0.006**
		N06DX01	*Memantine*	5.5 (25)	5.6 (12)	5.4 (13)	0.921
Other psychostimulants 14.3% (65)	Other psychostimulants, (N06BX)	N06BX03	*Piracetam*	8.4 (38)	9.8 (21)	7.1 (17)	0.288
		N06BX18	*Vinpocetine*	7.0 (32)	8.4 (18)	5.8 (14)	0.279
Antipsychotics 46.4% (211)	Atypical antipsychotics (N05A)	N05AH03	*Olanzapine*	4.0 (18)	1.4 (3)	6.2 (15)	**0.008**
		N05AH04	*Quetiapine*	15.4 (70)	15.0 (32)	15.8 (38)	0.810
		N05AX08	*Risperidone*	7.3 (33)	6.1 (13)	8.3 (20)	0.361
	Typical antipsychotics (N05A)	N05AL01	*Sulpiride*	1.1 (5)	0.5 (1)	1.7 (4)	0.377
		N05AL03	*Tiapride*	2.2 (10)	2.3 (5)	2.1 (5)	1.000
		N05AD01	*Haloperidol*	7.5 (34)	7.5 (16)	7.5 (18)	0.997
		N05AA02	*Levomepromazine*	1.3 (6)	1.4 (3)	1.2 (3)	1.000
		N05AA03	*Promazine*	7.9 (36)	9.3 (20)	6.6 (16)	0.286
		N05AB10	*Perazine*	10.1 (46)	12.1 (26)	8.3 (20)	0.174
		N05AF01	*Flupentixol*	0.4 (2)	–	0.8 (2)	0.501
		N05AF013	*Chlorprothixene*	0.9 (4)	0.5 (1)	1.2 (3)	0.626
		N05AF05	*Zuclopenthixol*	0.7 (3)	1.4 (3)	–	0.103
Anxiolytics 28.4% (129)	Anxiolytics (N05B)	N05BA01	*Diazepam*	2.0 (9)	3.3 (7)	0.8 (2)	0.090
		N05BA04	*Oxazepam*	0.4 (2)	0.9 (2)	–	0.221
		N05BA06	*Lorazepam*	0.9 (4)	0.9 (2)	0.8 (2)	1.000
		N05BA12	*Alprazolam*	1.1 (5)	0.5 (1)	1.7 (4)	0.377
		N05BB01	*Hydroxizine*	20.2 (92)	18.7 (40)	21.6 (52)	0.444
	Hypnotics and sedatives (N05C)	N05CD02	*Nitrazepam*	0.7 (3)	0.9 (2)	0.4 (1)	0.603
		N05CD04	*Estazolam*	3.1 (14)	3.3 (7)	2.9 (7)	0.821
		N05CD06	*Lormetazepam*	0.2 (1)	–	0.4 (1)	1.000
		N05CD07	*Temazepam*	0.2 (1)	–	0.4 (1)	1.000
		N05CD08	*Midazolam*	0.2 (1)	–	0.4 (1)	1.000
		N05CF01	*Zopiclone*	0.9 (4)	–	1.7 (4)	0.126
		N05CF02	*Zolpidem*	0.9 (4)	0.5 (1)	1.2 (3)	0.626

Chi^2^ test used. Values for which *p *< 0.05 are highlighted in bold

^a^*ATC* the Anatomical Therapeutic Chemical Classification

### Factors associated with prescribing medicines from certain therapeutic classes

Upon logistic regression analysis (Table 3), we found that RH residents had 2.88 times higher chance to be administered ADM (donepezil, rivastigmine or memantine) compared to patients in NHs. Moreover, AD (OR = 4.378, 95%CI 2.173–8.823) and hallucinations or delusions (OR = 2.244, 95%CI 1.170–4.306) significantly increased the chance of taking these drugs.

**Table 3:** Factors associated with prescribing psychotropics from certain ATC classes in LTC residents with cognitive impairment—the results of univariable and multivariable regression analyses

	Univariable logistic regression model		Multivariable logistic regression model	
	OR (95% CI)	*p* value	OR (95% CI)	*p* value
Anti-dementia medicines				
Facility type^a^ (NH, ref.)	2.365 (1.318–4.243)	**0.004**	2.875 (1.491–5.542)	**0.002**
Gender (female, ref.)	0.533 (0.274–1.038)	0.064	0.882 (0.412–1.888)	0.747
Age	1.023 (0.998–1.048)	0.071	1.012 (0.983–1.042)	0.426
Alzheimer’s disease (No, ref.)	6.085 (3.232–11.457)	** < 0.001**	4.378 (2.173–8.823)	** < 0.001**
Hallucinations or delusions (No, ref.)	2.651 (1.523–4.614)	**0.001**	2.244 (1.170–4.306)	**0.015**
Wandering (No, ref.)	2.243 (1.256–4.006)	**0.006**	1.154 (0.587–2.265)	0.678
Mild CI^b^ (ref.)	1		1	
Moderate CI^b^	1.647 (0.791–3.430)	0.182	1.261 (0.557–2.854)	0.578
Severe CI^b^	1.485 (0.781–2.824)	0.227	1.421 (0.687–2.940)	0.343
*Constant*			0.014 (0.001–0.169)	**0.001**
Other psychostimulants				
Facility type^a^ (NH ref.)	0.676 (0.399–1.147)	0.147	0.537 (0.298–0.967)	**0.038**
Gender (female ref.)	0.667 (0.360–1.234)	0.197	0.800 (0.408–1.569)	0.516
Age (78 years)	1.022 (0.998–1.047)	0.072	0.980 (0.947–1.015)	0.252
Facility type x Age	–	–	1.064 (1.011–1.121)	**0.018**
Mild CI^b^ (ref.)	1		1	
Moderate CI^b^	1.689 (0.852–3.348)	0.133	1.433 (0.701–2.927)	0.324
Severe CI^b^	1.135 (0.608–2.118)	0.692	0.767 (0.393–1.497)	0.437
Other dementia (No, ref.)	1.829 (1.023–3.270)	**0.042**	1.873 (1.007–3.485)	**0.047**
Socially inappropriate behavior (No, ref.)	1.376 (0.797–2.375)	0.252	1.428 (0.808–2.525)	0.220
* Constant*			0.137 (0.065–0.292)	** < 0.001**
*Effect of interaction: facility type (RH) × age: 1.064 × 0.980 = 1.043. It means a 4% higher chance*				
Typical antipsychotics				
Facility type^a^ (NH ref.)	0.696 (0.461–1.050)	0.084	0.761 (0.464–1.246)	0.277
Gender (female ref.)	0.951 (0.607–1.491)	0.826	0.898 (0.534–1.511)	0.686
Age	0.981 (0.965–0.998)	**0.026**	0.975 (0.957–0.994)	**0.009**
Mild CI^b^ (ref.)	1		1	
Moderate CI^b^	1.054 (0.596–1.863)	0.856	1.002 (0.551–1.822)	0.995
Severe CI^b^	1.374 (0.961–2.192)	0.183	1.122 (0.661–1.904)	0.669
Psychiatric disease^c^ (No, ref.)	1.576 (0.672–3.700)	0.296	1.526 (0.625–3.729)	0.354
Agitation (No, ref.)	1.587 (1.026–2.453)	**0.038**	1.296 (0.769–2.185)	0.330
Regular contact with family (No, ref.)	1.223 (0.803–1.864)	0.349	1.091 (0.671–1.774)	0.725
Chair preventing rising^e^ (No, ref.)	1.710 (0.950–3.081)	0.074	1.394 (0.708–2.743)	0.337
Wandering (No, ref.)	1.391 (0.865–2.239)	0.174	1.269 (0.745–2.163)	0.380
Resistance to care (No, ref.)	1.612 (1.051–2.473)	**0.029**	1.355 (0.812–2.261)	0.246
* Constant*			2.190 (0.430–11.165)	0.346
Atypical antipsychotics				
Facility type^a^ (NH ref.)	1.387 (0.898–2.142)	0.140	1.428 (0.893–2.282)	0.137
Gender (female ref.)	0.951 (0.593–1.523)	0.833	1.318 (0.773–2.247)	0.310
Age	1.027 (1.008–1.048)	**0.007**	1.032 (1.009–1.055)	**0.006**
Mild CI^b^ (ref.)	1		1	
Moderate CI^b^	0.874 (0.484–1.581)	0.657	0.745 (0.393–1.412)	0.367
Severe CI^b^	1.041 (0.642–1.688)	0.872	1.195 (0.697–2.049)	0.517
Psychiatric disease^c^ (No, ref.)	1.299 (0.524–3.220)	0.572	1.250 (0.479–3.258)	0.648
Delusions (No, ref.)	1.755 (1.092–2.820)	**0.020**	2.082 (1.199–3.613)	**0.009**
Wandering (No, ref.)	1.609 (0.987–2.623)	0.057	1.523 (0.882–1.630)	0.131
Resistance to care (No, ref.)	0.674 (0.417–1.088)	0.106	0.451 (0.258–0.788)	**0.005**
*Constant*			0.019 (0.003–0.129)	** < 0.001**
Anxiolitics				
Facility type^a^ (NH ref.)	1.123 (0.746–1.692)	0.578	1.115 (0.697–1.784)	0.649
Gender (female ref.)	1.023 (0.656–1.596)	0.920	1.027 (0.620–1.703)	0.917
Age	1.006 (0.989–1.024)	0.470	1.008 (0.989–1.028)	0.390
Mild CI^b^ (ref.)	1		1	
Moderate CI^b^	0.573 (0.320–1.026)	0.061	0.528 (0.289–0.966)	**0.038**
Severe CI^b^	0.893 (0.568–1.405)	0.626	0.796 (0.483–1.312)	0.371
Agitation (No, ref.)	1.558 (1.011–2.401)	**0.044**	1.452 (0.882–2.389)	0.142
Hallucinations (No, ref.)	1.616 (0.954–2.736)	0.074	1.584 (0.888–2.826)	0.119
Regular contact with family (No, ref.)	0.774 (0.512–1.169)	0.223	0.774 (0.485–1.233)	0.280
Verbal abuse (No, ref.)	1.972 (1.172–3.317)	**0.010**	1.358 (0.820–2.252)	0.235
*Constant*			0.212 (0.042–1.079)	0.062
Hydroxizine				
Facility type^a^ (NH ref.)	1.197 (0.755–1.897)	0.445	0.976 (0.559–1.703)	0.931
Gender (female ref.)	0.968 (0.586–1.599)	0.899	0.957 (0.534–1.715)	0.884
Age	1.012 (0.992–1.032)	0.242	1.016 (0.993–1.039)	0.187
Mild CI^b^ (ref.)	1		1	
Moderate CI^b^	0.650 (0.335–1.263)	0.204	0.486 (0.181–1.302)	0.151
Severe CI^b^	1.039 (0.626–1.724)	0.883	0.465 (0.188–1.150)	0.097
Other dementia (No, ref.)	0.825 (0.519–1.312)	0.417	0.735 (0.443–1.218)	0.232
Hallucinations (No, ref.)	1.637 (0.921–2.910)	0.093	1.693 (0.880–3.256)	0.115
Aggressive behaviour (ABS)^d^ (No, ref.)	1.467 (0.922–2.333)	0.106	0.665 (0.288–1.539)	0.341
Chair preventing rising^e^ (No, ref.)	0.543 (0.237–1.244)	0.149	0.444 (0.177–1.116)	0.084
Regular contact with family (No, ref.)			0.577 (0.337–0.988)	**0.045**
Mild CI × Aggressive behaviour (ABS)	-	-	1	
Moderate CI × Aggressive behaviour (ABS)	-	-	2.528 (0.611–10.454)	0.200
Severe CI × Aggressive behaviour (ABS)	-	-	4.591 (1.377–15.300)	**0.013**
*Constant*			0.144 (0.021–0.986)	**0.048**
*Effect of interaction: CI (severe) × aggressive behaviors (yes) (ABS): 0.465 × 4.591 = 2.5998. It means a 2.6 higher chance*				

Values for which *p* < 0.05 are highlighted in bold

^a^Facility type (*NH* nursing home, *RH* residential home)

^b^Cognitive performance scale (CPS): mild cognitive impairment (CPS = 2), moderate cognitive impairment (CPS = 3–4), and severe cognitive impairment (CPS = 5–6)

^c^Psychiatric diseases except depression and dementia

^d^Aggressive Behaviour Scale (ABS): no signs of aggressive behavior (0), any sign of aggressive behaviors (1–12)

^e^Chair preventing rising: armchair with strips to prevent uncontrolled rising or falling

On the contrary, prescribing of OP (piracetam or vinpocetine) was 87% more likely in patients with dementia other than AD and almost twice less likely in the residents of RHs. Moreover, in RHs, as the age of a resident increases by 1 year, the chance they are administered OP increases by 4% (the method of calculating the interaction effect is shown in Table 3).

We found that dementia other than AD (OR = 1.520, 95%CI 1.016–2.273) and use of chair preventing rising (to protect uncontrolled getting up and fall) (OR = 2.466; 95%CI 1.272–4.780) were associated with an increased use of antipsychotics in general. However, factors associated with use of typical or atypical antipsychotics differed. T-APM were administered to 27.9% of residents, but none of the analyzed factors were associated with their prescribing, except older age which decreased odds ratio (OR = 0.975; 95%CI 0.957–0.994). On the contrary, the chance of being treated with A-APM (used by 24.2% residents) was significantly higher in older patients (OR = 1.032; 95%CI 1.009–1.055) and individuals with delusions (OR = 2.082, 95%CI 1.199–3.613). The residents who resisted to care had a lower risk (by 54.9%) to be administered A-APM, but higher, although not significant risk of receiving T-APM.

Residents with moderate CI were nearly twice less likely (by 47.2%) to receive anxiolytics compared to those with mild CI. Hydroxyzine was the most often used (71.3%) among anxiolytics; therefore, we conducted multivariable regression analysis for this single drug. Severe CI (compared to mild CI) increased the chance of receiving this drug 2.6 times in patients presenting aggressive behavior disorders (assessed with ABS). The use of physical restraints (especially full bed rails) was high (Table 1). Therefore, we conducted logistic regression analysis using them all together as one variable and each of them separately, but did not prove their association with prescribing of any of analyzed psychotropics class.

### Psychotropic drugs prescribing patterns in LTC residents

Next, we checked whether there was a correlation between the use of selected classes of psychotropic drugs and the type of facility, level of CI and resident’s age. By applying MCA, we identified two dimensions (axes), which explained a 37.06% improved estimate of the inertia among the seven factors (CI level, resident’s age, type of facility, use of drugs from ATC classes: A-APM, T-APM, OP and anxiolytics). For the first factorial axis (axis *X*), the principal discrimination measures were associated with LTCI type (0.478) and CI level (0.434). For the second axis (axis *Y*), the discrimination measures were those mostly associated with using or not A-APM (0.470). In the result of MCA, we identified three groups of residents. The first group (marked in the green circle) consisted of the individuals with severe CI, more frequently aged 75–84 years, residing in NH, and taking OP treatment. The second group (marked with the gray circle) contained patients who more often received A-APM and anxiolytics but less often T-APM, and that way of treatment was applied mostly to the oldest individuals at the age 85 years or older. The third one (marked with the yellow circle) included individuals more often taking T-APM, and less frequently anxiolytics and A-APM, who were residents with moderate CI residing in NH (Fig. 1).

## Discussion

### Use of anti-dementia medicines (donepezil, rivastigmine, memantine) and other psychostimulants

In our study, the use of ADM was less prevalent in Polish LTCIs (13.4%) compared to US [24], where 30% of any type dementia residents were administered acetylcholinesterase inhibitors (AChEIs). It was a little higher than in the European SHELTER study (11.6% residents with severe CI), yet our study was conducted 5 years after SHELTER one, when prescribing of ADM has become more common. It has been improved in our country after including donepezil and transdermal rivastigmine on the “list of drugs for seniors 75+ ” allowing patients with AD diagnosis (ICD-coded G30) to receive these drugs free of charge, but patients with a diagnosis of other dementia (ICD-codes: F01, F02, F03) could not get any discount. Therefore, in our study, patients with dementia other than AD were more likely to receive cerebral vasodilators and nootropic drugs (e.g. piracetam, vinpocetine), despite these medicines not being recommended in light of current research, because they are ineffective in both vascular dementia (VaD) [25, 26] and Lewy body dementia (DLB) [27]. These findings indicate that prescribing of ADM may be suboptimal especially in mild-to-moderate stages of dementia. More in depth study is needed to shed more light on potential reasons of that, e.g. financial limitations, administrative obstacles, or simply lack of knowledge on diagnosis and treatment of dementia.

### Use of typical and atypical antipsychotic drugs

Based on a systematic review by Janus et al. [28], the rates of use of APM in Western and Nordic European NHs range from 12 to 59% with the highest in Austria, Ireland and Belgium. In the SHELTER study conducted in 57 NHs in 8 European countries, 26.4% of all NH residents [29], and 35.6% of residents with severe CI [30] were treated with antipsychotics (in 2010). In the US, this percentage was much lower than in Europe with tendency to decline—in 14.6% of all residents, and 24.8% of dementia patients in NHs (in the year 2014) [31]. In light of these data, our results look dramatic—antipsychotics were prescribed to 46.4% of RH and NH residents with CI. There was only one other study on use of these medicines in Poland—it showed similarly high rates of antipsychotics use (43.4%) in NH residents with moderate and severe dementia (in 2013) [32]. During the last decade, the use of A-APM has gradually been replacing typical neuroleptics, nevertheless number of residents treated with T-APM is still very high (27.9% in our study), even though this class of medicines should be avoided due to extrapyramidal and cholinolytic side effects. Multivariable regression analysis showed that prescribing A-APM is more probable in older residents, and when delusions appear. In contrary, there was no factor increasing the odds of T-APM use. The last finding may suggest that there is no clear pattern of prescribing of these drugs, which may mean that physicians in LTCIs do not follow clinical recommendations.

### Use of anxiolytics

Compared to SHELTER study (where 36.0% of residents used benzodiazepines), the use of all anxiolytics in our LTCIs was lower (28.4% residents) with definitively lower usage of long acting benzodiazepines (4.4% of residents with CI) and hypnotics (short acting benzodiazepines and z-drugs in 6.2% of CI residents). However, 20.2% of studied residents (71.3% of all individuals taking any anxiolytic drug) received hydroxyzine, which according to Beers criteria should be avoided in older patients, especially in AD, dementia or other CI. We showed that aggressive behaviors in patients with severe CI might increase the risk of administering this medicine 2.6 times, despite the fact that it is a potentially inappropriate drug due to highly anticholinergic effects causing risk of confusion and cognitive decline [33]. It is worth noting that regular contact with family might reduce that risk by almost half.

### Use of physical restraints

There is robust literature that physical restraints are measures involving deprivation of liberty, which are associated with deaths and suppression in quality of life. Nevertheless, in our study, the use of restrictive devices as full bed rails was very common (57.1%), which is probably caused by the general belief of the staff that these are means of protecting the bedridden patients from falling out of bed. The use of other physical restraints was much less frequent (5.4% trunk restraints, and 12.3% chair preventing rising). However, taking into account that each use of them is strictly regulated and very limited by law, these proportions should be evaluated as relatively high.

We thought that pharmacological and physical restraints may be used together or interchangeably in dementia residents presenting NPS, but statistical analysis did not confirm that. We had conducted analysis including use of each type of physical restraints (the full bed rails, trunk restraints, chair preventing rising) separately and all together as one variable in the logistic regression models, however, we did not find any significant association between use of them and any of psychotropic class.

### Drugs prescribing practice in the LTC residents with cognitive impairment

As a result of the logistic regression analysis, we have received a clear message that Alzheimer’s disease is an independent predictor of ADM use, while other dementia increases the risk of prescribing OP (nootropic drugs). However, the level of CI had no impact on use of specific psychotropic drugs with an exception for anxiolytics (odds for their use was lower in moderate CI). In contrary, the presence of psychotic symptoms or aggressive behaviors increased the use of some of psychotropics. Brimelow et al. [34] also reported that agitation and psychotic symptoms in residents with dementia increased the prescribing of psychotropic medicines more than twice. Thus, it seems that the presence of certain symptoms is the main trigger to prescribe these drugs, not the diseases themselves.

In addition to the regression analysis, which provided us with information about determinants of use of certain drug classes separately, we examined if there are some classes of psychotropic drugs used together (or not). As a result of MCA we have gained better insight into how the residents with mild, moderate and severe CI are treated in LTCIs. We found that taking A-APM was often combined with using anxiolytics, but not T-APM, and it applied more often to the oldest residents (aged 85 years and older) (a gray circle, Fig. 1). In opposite, the residents who were administrated with T-APM, more often had moderate CI, and less frequently received A-APM and anxiolytics (a yellow circle, Fig. 1). In turn, taking OP was rather correlated with residing in NH, severe CI and being aged 75–84 years (a green circle, Fig. 1). Contrary to that, having mild CI and residing in RH was not related to more frequent taking of any psychotropic medications.

## Strengths and limitations

This is an epidemiological cross-sectional study, which in contrary to longitudinal study design cannot explain cause-effect relation between symptoms and drugs use. However, it is worth highlighting, that this is the first national research of the CI residents’ treatment in a country-representative sample of NHs and RHs in Poland conducted with use of a tool allowing comparisons with other countries in Europe and US. It showed some differences compared with other countries (lower use of anxiolytics and much higher use of antipsychotics), as well as revealed factors associated with taking these drugs. We focused on associations between resident characteristics and use of drugs from certain classes, and found that physicians taking care for LTC residents did not follow clinical recommendations for CI treatment, properly. We found the differences of prescribing practices between GPs providing care in RHs and physicians employed in NHs, however, due to lack of information about their specialties we could not conduct more in depth analysis to find if their knowledge and experience had impact on the type of treatment.

This paper points out the main treatment issues, which should be corrected to assure good quality of care. It provides some prompts for education of the physicians working in LTCIs, who often have different specialization background (not necessarily geriatrics). The rate of geriatricians in our country is one of the lowest in EU (0.06 per 1000 persons at age 65 and over), and most of them work in acute geriatric wards. Therefore, physicians working in LTC facilities, both GP and other specialists, definitely need training to improve their prescribing practice.

## Conclusions

In our study, we examined factors associated with prescribing ADM, A-APM, T-APM, anxiolytics and OP in the individuals with CI residing in LTCIs. We found that use of ADM was less frequent than in US and in other countries. Patients with AD had a higher chance of receiving ADM, while patients with other dementia—OP. Almost half of LTC residents with CI received antipsychotics, which is significantly higher than in other European and North American countries. In contrary, use of anxiolytics was much lower. We found that some NPSs (delusions, aggressive behavior), were significantly associated with higher use of some psychotropics. Moreover, we have observed specific prescribing practices correlated with residents’ age, CI level and facility type. We found that oldest residents more often used A-APM (compared to T-APM) and anxiolytics, while NH residents aged 75–84 with severe CI more often were prescribed OP. In contrast, the residents with moderate CI more often resided in NHs and were administered with T-APM.

Our analyses confirmed that there are still many LTC residents who receive medications that are not recommended or even contraindicated in dementia (e.g. T-APM and hydroxyzine). Despite existing clinical recommendations for treatment of CI and NPSs, the physicians taking care for LTC residents do not follow them properly. Hence, more attention should be given to motivate physicians to change their prescribing practices to provide residents with adequate and effective treatment.

## Electronic supplementary material

Below is the link to the electronic supplementary material.

Supplementary file1 (DOCX 26 kb)

## Abbreviations

AChEI: Acetylcholinesterase inhibitors
AD: Alzheimer’s disease
ADL: Activities of daily living
ADM: Anti-dementia medicines
APM: Antipsychotic medicines
ATC: The anatomical therapeutic chemical classification
A-APM: Atypical antipsychotic medicines
CI: Cognitive impairment
CMS: The Centers for Medicare and Medicaid Services
CPS: Cognitive performance scale
DLB: Lewy body dementia
ICD-code: International classification of diseases
LE: Life expectancy
LTC: Long-term care
LTCI: Long-term care institution
MCA: Multiple correspondence analysis
NH: Nursing home
NHF: National Health Fund
NMDA: The *N*-methyl-d-aspartate receptor antagonist
NPSs: Neuropsychiatric symptoms
OP: Other psychostimulants (i.e. medicines improving blood perfusion in the brain, but not indicated in dementia—N06BX, N07CA)
RH: Residential home
SHELTER: Services and Health for Elderly in Long-TERm care (SHELTER)
T-APM: Atypical antipsychotic medicines
VaD: Vascular dementia
