# Supplementary material for: Factors associated with drug prescribing practices in long-term care patients with cognitive impairment
Source: Eur Geriatr Med. 2020 May 25;11(5):761–75. doi: 10.1007/s41999-020-00331-0 (PMC7550298; doi:10.1007/s41999-020-00331-0)
Supplement: Supplementary file 1 — Supplementary file1 (DOCX 26 kb) [file 41999_2020_331_MOESM1_ESM.docx]

**SUPPLEMENTARY MATERIAL**

**Factors associated with drug prescribing practices in long-term care patients with cognitive impairment**

*Violetta Kijowska MPH^1^, Ilona Barańska, MPH^1^, Katarzyna Szczerbińska MD, PhD ^1^*

*(1) Unit for Research on Aging Society, Department of Sociology of Medicine, Jagiellonian University Medical College, Krakow, Poland*

**Corresponding author:**

Katarzyna Szczerbińska, MD, PhD

Unit for Research on Aging Society, Department of Sociology of Medicine

Jagiellonian University Medical College

Kopernika 7a Street

31-034 Krakow, Poland

[katarzyna.szczerbinska@uj.edu.pl](mailto:katarzyna.szczerbinska@uj.edu.pl)

+48 602-240-016

**ORCID - 0000-0002-0004-3858**

**Long-term care services in Poland**

The long term care (LTC) services for dependent older people in Poland are delivered through two sectors: the health care system and the social care system.

Nursing home (NH) in Poland reminds the most a skilled nursing facility (according to the classification proposed by Stanford et al.), with the main aim to avoid hospitalization of community dwelling patients or facilitate early hospital discharge (Stanford et al. 2015). NH is mainly managed by a physician as a medical director, and provides 24-hour a day and 7 days a week nursing and medical care by medical staff, including on-site employed doctors (with different specialties), nurses (specialists in long-term care nursing), physiotherapists, occupational therapists, social workers and psychologists. Costs of health care are funded by health insurance, and co-payment of 70% of the patient's monthly income applies only to the costs of food and accommodation. NH residents are provided with necessary medicines, orthopedic items, auxiliary agents (e.g. diaper pants), diagnostic tests, enteral and parenteral nutrition free of charge.

The residential home (RH) is a facility primarily intended for those who require assistance with ADLs and instrumental activities of daily living (IADLs) (Stanford et al; JAMDA). Its main aim, considering that the patient stays there permanently, is to provide a supportive and safe, homey environment while assisting the resident in maintaining functional status for as long as possible. The RH assures around-the-clock personal care performed by the on-site employed care assistants who are trained in ADL and IADL assistance, physiotherapist, occupational therapists, social workers, and psychologists. According to the Central Statistic Office data, only 13% nurses and 2% physiotherapists is employed in residential homes. Family doctor (an off-site physician) visits the resident in the RH or the resident is admitted to the outpatient clinic. The RH resident is treated as the other community-dwelling people, so the access to medical care (i.e. specialists, hospital) is based on a family doctor’s referral. RHs are financed by general taxation. Funding is divided amongst four payers, where the state is estimated to cover 75% of the overall cost of a residential home. This is supplemented by co-payments from the resident (max.70% of their monthly income), the family and the local governmental budget. The RH resident is charged with bearing the costs of purchasing medicines and hygiene products.

***Supplementary Table 1*** *Stationary LTC settings in Poland involved into the study – a comparison between NH and RH*

|  | **Nursing homes – NH**  (in health care sector) | **Residential homes – RH** (in social care sector) |
| --- | --- | --- |
| Types | Care and treatment facilities (ZOL)  Nursing and care facilities (ZPO) | Residential care homes for chronically ill persons (DPS). |
| The main goal | Providing care for chronically ill people to prepare them to come back home. | Providing care for older or chronically ill persons who due to ADL dependency and lack or insufficient family care are not able to stay at home. So they stay in RH usually forever. |
| ALOS – average length of stay | 5-6 months | Years |
| Admission criterion | Barthel Index score less than 40 pts and medical indications for care e.g. tube feeding, wound care, bedsores, dwelling catheter, stoma care, PEG. | ADL dependency or chronic illness, and lack or insufficient family support so patient needs continuous supervision and care, and is unable to stay at home alone. |
| Service profile | Ensuring round-the-clock medical and nursing care (for 24-hour a day for 7 days a week); physiotherapy and occupational therapy for 5 days a week, access to psychologist, dietician, as well to specialist physician’s consultancy. | Ensuring round-the-clock care provided by care assistants; nursing care during the day; physiotherapy and occupational therapy for 5 days a week; consultancy of other professionals (ex. psychologist, dietician, social worker), and educational services.  The residents are visited by a family doctor (GP) in the RH or are admitted to outpatient clinics by GP or specialist physicians for consultancy. |
| Staff | *Medical staff (on-site physicians and nurses specialized in long term care nursing*).  Doctors – full time 24/7 (on-site) Nurses – full time 24/7 (on-site) Physiotherapists Occupational therapists Psychologists  Dieticians | *Formal care assistants and other qualified staff* *who are employed on-site in the facility.*  Care assistants – full time 24/7 (on-site) Nurses (1:5 caregivers) (mainly off-site) Physiotherapist Occupational therapists Social workers Psychologists  Dieticians |
| Funding | Health care services paid based on contract with National Health Fund (NFZ) from health insurance. | Paid from governmental budget (financed through taxes). |
| Co-payment rules | An obligatory co-payment of 70% of the patient's monthly income applies only to the costs of food and accommodation. | An obligatory co-payment of 70% of the patient's monthly income for residential and home care services. A partial family co-payment for accommodation.  Additionally, the resident has to pay charges for medicines and supportive devices based on the same rules as the other community-dwelling people. |
| Drugs prescription | Drugs prescribed at any time by the physician employed in the facility, immediately available for patient from the local pharmacy. The prescription can be changed at any time. | Drugs prescribed exactly in the same way as for community dwelling patients. A prescription for reimbursed drugs can be issued by a doctor (GP or specialist physician) and dentist; nurse and midwife (a limited list of drugs). Drugs are bought in a pharmacy (out of RH). |
| Distribution of drugs | Medicines are distributed and delivered everyday by nurses. | Medicines are distributed everyday by nurses to dispensary boxes and delivered by care-assistants. |
| Costs of drugs use | All the drugs and any treatment is for free for the resident. Such costs are paid by the NH budget, and covered by health insurance. | The resident is obliged to cover the costs of purchasing drugs. The following levels of reimbursement are in use: 100%, 30%, and the lump sum (about €0.8), on the basis of a prescription issued by an authorized person (physician, nurse). The generic substitution of drugs is desired but not mandatory. A governmental program “75+”, entitles people aged 75 or older to get a free supply of some medicines, food for special nutritional uses and medical devices from the list of reimbursed drugs. |
|  | *No matter where physicians are working they have some guidelines when they can prescribe drugs, when – in which condition this drug can be prescribed for what payment 0-100%, which is regulated by the National Health Fund.* | |
| Access to physicians | Medical doctor in NH is available every day on-site. | GP is coming to RH once a week on a regular base and is visiting the residents (selected by RH staff), depending on their conditions. |
| Access to specialized medical care | Access to specialists and to hospital on the basis of written referral from the physician employed in the facility. | Access to medical care (i.e. family doctors, specialists physicians, hospital and other healthcare services) is according to the rules applied to the older people living in the community. |
| Access to medical and assistive devices | NH residents are provided with necessary medicines, orthopedic items, auxiliary agents (e.g. diaper pants), diagnostic tests, enteral and parenteral nutrition for free. | The RH resident is charged with bearing the costs of purchasing medicines and hygiene products. |

References:

1. Golinowska S., Sowa A. Działania samorządów lokalnych w opiece i integracji niesamodzielnych osób starszych, Institute of Labour and Social Studies (IPiSS) report commissioned by the Ministry of Labour and Social Policy, Warszawa 2010
2. Sanford AM, Orrell M, Tolson D, Abbatecola AM, Arai H, Bauer JM, et al. An International Definition for “Nursing Home.” J Am Med Dir Assoc 2015;16:181–4. doi:10.1016/j.jamda.2014.12.013.
3. Social assistance, child and family services in 2017. Statistics Poland, Social Surveys Department, Warsaw 2018.
